# Frequency and clinical significance of prostatic involvement in men with febrile urinary tract infection: a prospective observational study

**DOI:** 10.12688/f1000research.24094.3

**Published:** 2020-11-10

**Authors:** Thayyil Shahilal Arjunlal, Surendran Deepanjali, Ramanitharan Manikandan, Rajappa Medha

**Affiliations:** 1 Department of Medicine, Jawaharlal Institute of Postgraduate Medical Education and Research (JIPMER), Dhanvantri Nagar, Puducherry, 605006, India; 2 Department of Urology, Jawaharlal Institute of Postgraduate Medical Education and Research (JIPMER), Dhanvantri Nagar, Puducherry, 605006, India; 3 Department of Biochemistry, Jawaharlal Institute of Postgraduate Medical Education and Research (JIPMER), Dhanvantri Nagar, Puducherry, 605006, India

**Keywords:** urinary tract infections, prostate-specific antigen, men, prostatitis, antibiotic treatment duration

## Abstract

**Background**: Frequent asymptomatic involvement of the prostate has been demonstrated in men with febrile urinary tract infection (fUTI). In view of this, men with fUTI are often given a longer duration of antibiotic treatment; however, evidence to support this is limited.

**Methods**: We prospectively studied adult men with fUTI admitted under the Department of Medicine in a tertiary care hospital in southern India.  fUTI was defined as fever of ≥38°C with at least one symptom/sign of UTI and pyuria, requiring hospitalization. We estimated serum total prostate-specific antigen (PSA) levels at enrollment, one month and three months after treatment completion. We assessed prostatic volume by transrectal ultrasonography (TRUS) and estimated the serum high sensitivity C-reactive protein (hs-CRP) levels at baseline and after three months.

**Results**: We enrolled 64 men (median [IQR] age 53 [45-60] years); 50 patients completed follow-up. At baseline, 24 (38%) of 64 patients had elevated serum PSA values compared to age-specific upper limit. The median (IQR) serum PSA level was 2.15 (1.18-3.02) ng/mL and median (IQR) serum hs-CRP level was 2.23 (1.85-2.74) mg/dL (N=64). At three months, serum PSA levels decreased by ≥25% in 47 (94%) of 50 patients. The median (IQR) of prostatic volume was 25.4 (18.9-34) mL at baseline (N=64), and ≥10% decrease in prostatic volume was observed in 24 (48%) of 50 patients at three months. The change in the serum PSA levels did not correlate with clinical findings like prostatic tenderness or with prostatic volume changes. Further, serum PSA levels did not correlate with hs-CRP levels. On follow-up, seven patients had lower urinary tract symptoms; only one of them had recurrent fUTI.

**Conclusions**: Asymptomatic prostatic involvement, although common in men with fUTI, does not seem to influence the treatment outcomes.

## Introduction

Urinary tract infections (UTIs) in men are generally considered to be complicated UTIs because of increased prevalence of underlying structural and functional abnormalities ^1^. One such abnormality is the possibility of involvement of the prostate gland during an episode of UTI. Symptomatic involvement of the prostate gland by acute bacterial infection, known as acute bacterial prostatitis (ABP), classically presents with fever and systemic symptoms along with pelvic pain and infravesical obstruction ^2^. However, even in the absence of prominent voiding and storage symptoms, subclinical involvement of the gland is possible in men with febrile UTI (fUTI). A study of Swedish men with fUTI provided the major evidence for this, in the form of increased serum prostate-specific antigen (PSA) levels and prostatic volume during an episode of UTI ^3^. Another study using ^111^indium-labelled leukocyte scintigraphy found that, although often clinically unrecognized, the prostate was involved in most cases of fUTI and acute pyelonephritis in men ^4^.

Differentiating fUTI with subclinical prostatic involvement from classical ABP is important. First, some experts recommend that antibiotic treatment in men with fUTI should not only sterilize the urine but also achieve sufficient concentrations in the prostate. Fluoroquinolones and co-trimoxazole were recommended as optimal choices to achieve this aim ^5, 6^. If the increased levels of PSA are truly indicative of ABP, implementing this guidance would be pragmatically challenging in settings where antimicrobial resistance, especially to fluoroquinolones, is very common among uropathogens and other appropriate oral options are not available ^7^. Second, while it is generally agreed that ABP requires antibiotic treatment for at least two to four weeks to prevent chronic prostatitis ^8^, it is unclear whether subclinical prostatic involvement would also necessitate a longer treatment. We, therefore, conducted the present study to answer the following research questions — i) What is the frequency of prostatic involvement in men with fUTI, and ii) Is prostatic involvement associated with recurrence of UTI?

## Methods

### Ethical statement

The study protocol was reviewed and approved by the Institute Ethics Committee (Human Studies) of Jawaharlal Institute of Postgraduate Medical Education and Research, Puducherry (JIP/IEC/2016/29/970). Written informed consent was obtained from all study participants.

### Study setting

We conducted a prospective observational study in the medical wards of Jawaharlal Institute of Postgraduate Medical Education and Research (JIPMER), a tertiary care hospital in Southern India, during July 2016 and May 2018. During this study period, we screened all consecutive male patients aged 18 years or over admitted under the Department of Medicine as suspected cases of fUTI for eligibility to participate in the study. We defined fUTI as documented fever of at least 38°C with at least one symptom or sign referable to the urinary tract such as dysuria, frequency, urgency, flank pain or renal angle tenderness. All patients had evidence of microscopic pyuria (>5 pus cells/hpf) or urine dipstick positive for leukocyte esterase. We excluded patients with catheter-associated UTI, urological procedures or surgery in the past four weeks, diagnosed with prostate carcinoma, or significant upper urinary tract obstruction evidenced by gross hydroureteronephrosis (HUN).

### Study procedure

After obtaining written informed consent, the investigator (TSA) performed a standardized clinical evaluation. A digital rectal examination (DRE) was done to look for prostatic enlargement, tenderness or bogginess. Blood samples were drawn within 48 hours of admission for serum PSA and high sensitivity C-reactive protein (hs-CRP) estimation, and serum was stored at -80°C for batched analysis. Since DRE may lead to an increase in serum PSA levels, blood samples were drawn before performing DRE. Serum PSA levels were estimated in duplicate using a two-site immune-enzymatic sandwich assay that uses a mouse monoclonal anti-PSA antibody (Cat. No. 37200, Hybritech Prostate Specific Antigen, Beckman Coulter, Fullerton, CA) as described for the National Health and Nutrition Examination Survey 2001–2002 ^9^. Serum hs-CRP was estimated in duplicate using a solid phase direct sandwich assay which uses a monoclonal antibody (Cat. No. CR120C, Calbiotech. Inc., El Cajon, CA) as per the manufacturer’s instructions. All patients underwent ultrasonographic examination of kidney, ureter and bladder. As soon as the patients became clinically stable, a transrectal ultrasound (TRUS) examination of the prostate and seminal vesicles was performed using Famio 8 SSA-530A (TOSHIBA), PVQ 641V 6 MHz probe by a urology senior resident. We collected data on the antibiotic regimen started, duration of therapy and clinical course during hospital stay.

At the first follow-up assessment after one month of treatment completion, serum PSA estimation was done in all patients. In those with persistent fever or urinary tract symptoms, urine dipstick leukocyte esterase test and microscopic examination were done. In those with evidence of significant pyuria, urine culture was repeated. At the second follow-up visit after three months of treatment completion, clinical evaluation and repeat measurements of serum levels of PSA and hs-CRP were done. TRUS was also repeated to re-assess the size of prostate and to assess resolution or persistence of inflammation. A baseline serum PSA level above 97.5th percentile of decade-specific serum PSA levels in healthy Indian men was considered to be elevated. We calculated this upper limit cut-off based on a previous study of serum PSA levels among 1300 healthy adult Indian men ^10^. We used the decade-specific mean and standard deviation (SD) of PSA values and calculated the 97.5 ^th^ percentile as mean + 1.96 SD. We defined prostatic involvement as per the criteria suggested by Ulleryd *et al.* ^3^. A reduction of serum PSA by >25% irrespective of the initial PSA level, and/or a decrease in prostatic volume by >10% between the acute phase and the follow-up after 3 months was taken as evidence of prostatic involvement.

### Sample size calculation

Assuming that 80% of patients would have evidence of prostatic involvement ^3^, 64 patients were required to estimate this proportion with 10% absolute precision.

### Statistical analysis

We summarized categorical variables as n (%) and continuous variables as mean±SD or median (IQR) as appropriate. We applied the Wilcoxon signed rank test to assess the change in serum PSA and serum hs-CRP levels at three months compared to baseline. We performed the Friedman test to analyze the trend of serum PSA at admission, one month and three months. We applied the Wilcoxon rank sum test to compare baseline serum PSA and change in serum PSA levels at three months between patients with and without clinical features suggestive of prostatic involvement. We tested the correlation between baseline serum PSA levels and fall in its levels by three months and that between baseline serum PSA levels and the change in serum hs-CRP levels by three months using Spearman’s rank correlation. All analyses were performed using the statistical software package Stata/IC 12.1 for Windows, StataCorp LP, College Station, Texas, USA. All tests were two-sided, and P <0.05 was considered statistically significant. We used GraphPad Prism version 8.3.0 for Windows, GraphPad Software, San Diego, California USA for graphical summaries.

## Results

**Figure 1.**
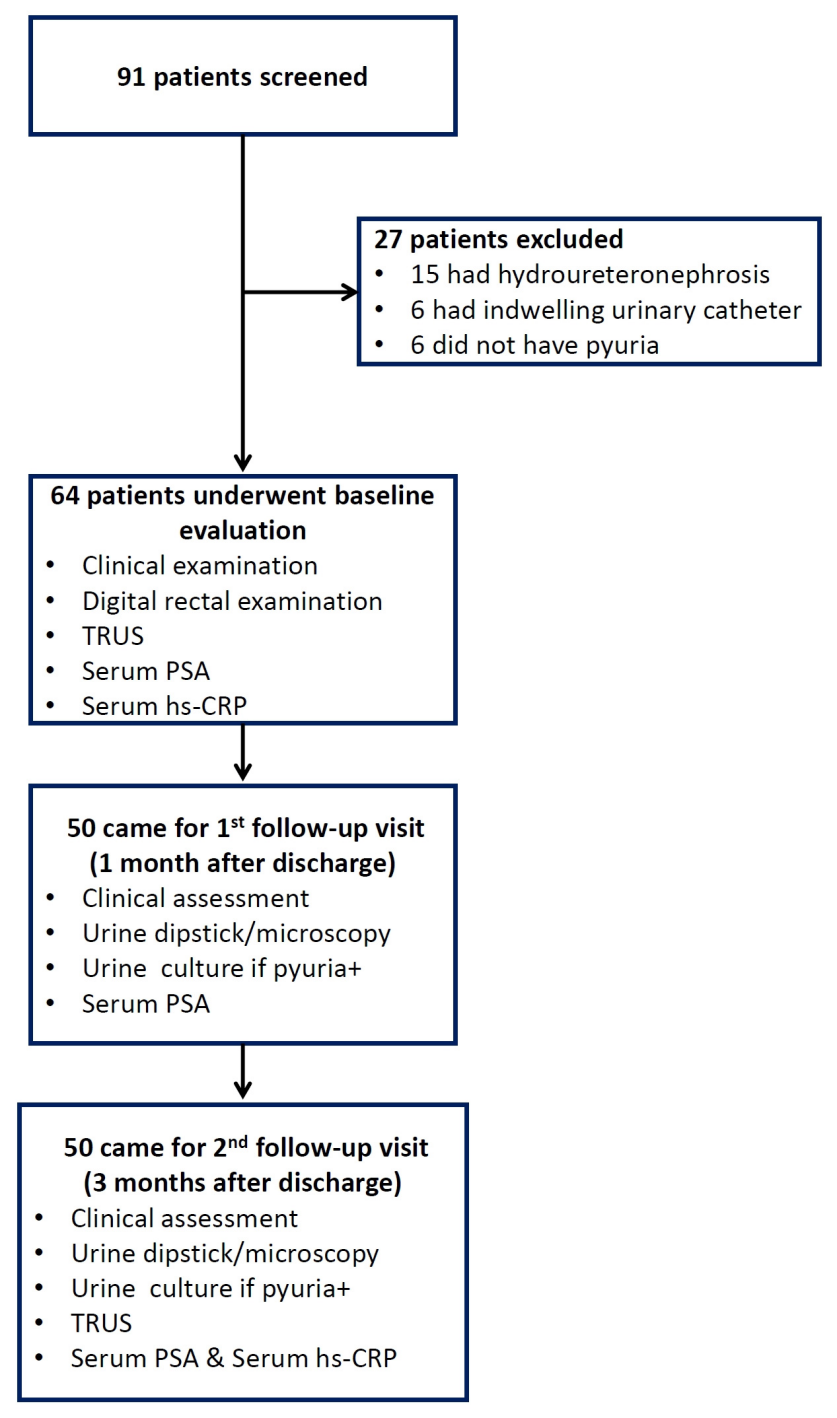
Flow of participants through the study. TRUS, transrectal ultrasound; PSA, prostate-specific antigen; hs-CRP, high sensitivity C-reactive protein.

Between July 2016 and May 2018, we screened 91 men with a diagnosis of fUTI and included 64 patients; 50 patients completed follow-up assessments at one month and three months. Figure 1 depicts the flow of subjects through the study. Clinical and laboratory characteristics of the study subjects at baseline are summarized in Table 1. Notably, 16(25%) of 64 patients had presented with obstructive urinary symptoms and 25 (39%) patients had prostatic tenderness on DRE.

**Table 1.** Clinical, imaging and laboratory features at admission.

Characteristic	Frequency (n=64)
Age in years, median (IQR)	53(45-60)
Past history of UTI, n(%)	6(9)
Hypertension, n(%)	7(10)
Diabetes, n(%)	23(36)
Chronic kidney disease	8(12)
Co-morbid conditions ^a^, n (%)	11(17)
**Clinical features**	
Fever, n(%)	64(100)
Dysuria, n(%)	64(100)
Frequency, n(%)	20(31)
Urgency, n(%)	2(3)
Hematuria, n(%)	1(1.5)
Lower abdominal pain, n(%)	52(81)
Painful ejaculation, n(%)	3(5)
Vomiting, n(%)	2(3)
Retention of urine, n(%)	16(25)
Renal angle tenderness, n(%)	37(58)
Hypotension, n(%)	6(9)
**Digital rectal examination findings**	
Normal, n(%)	34(53)
Tender and enlarged prostate, n(%)	8(12)
Tender prostate, n(%)	17(27)
Enlarged prostate, n(%)	5(8)
**Transabdominal ultrasound findings**	
Normal findings	13(20)
Cystitis	42(66)
Pelvicalyceal splitting	27(42)
Bilateral contracted kidneys	5(8)
Renal cysts	5(8)
Renal Stones	1(2)
Ependymal calcification	1(2)
Orchitis	1(2)
Congenital anomaly (duplex kidney)	1(2)
Mild hydroureteronephrosis	1(2)
**Laboratory parameters**	
Total leukocyte count (cells/µL), mean±SD	14599±11027
Serum creatinine at admission (mg/dL), median(IQR)	1.7(1.1-4.5)
Positive urine dipstick leukocyte esterase, n(%)	64(100)
Urine microscopy pus cells >5 cells/hpf	51(80)
Positive urine culture, n(%)	29(45)
Positive blood culture (N=31), n(%)	12(39)

a = co-morbid conditions include coronary artery disease, malignancy, chronic liver disease, cerebrovascular accident. UTI, urinary tract infection; IQR, interquartile range; SD, standard deviation; hpf, high-power field.

Mean duration of fever was 6.7±4.4 days; 15 (23%) of 64 patients had received antibiotics prior to study enrollment. Using a strict diagnostic criteria of fever, dysuria, urinary retention and prostatic tenderness on DRE, eight (12%) of 64 patients could be classified as cases of ABP. Urine culture was sent prior to the first dose of antibiotic after admission in 45 (70%) of 64 patients. *Eschericia coli* was the major uropathogen, isolated in 25 (86%) of these 45 patients.

### Antibiotic therapy

Empirical antibiotic regimens used in the study population (N=64) were ceftriaxone in 28 (44%), amikacin in 21 (33%), a combination of ceftriaxone and amikacin in seven (11%), cefaperazone/sulbactum in three (5%), a combination of piperacillin/tazobactum and amikacin in three (5%) patients and meropenem in one (2%) patient. A patient with *Candida tropicalis* fungemia and prostatic abscess was treated with fluconazole for 28 days. Modification of the empirical regimen based on susceptibility was done in six (9%) patients. Mean duration of antibiotic therapy was 8.6±3.6 days.

### Follow-up assessments and UTI recurrence

Of the 64 patients included, 50 patients came for first follow-up visit after one month of treatment completion. Among them, persistent lower urinary tract symptoms (LUTS) were present in seven (14%) patients. One of them (patient 1), who had fungal prostatic abscess at initial admission, gave a history of recurrent fever. He had significant microscopic pyuria and urine culture showed significant growth of *E. coli*, which was susceptible to amikacin. He was re-admitted and was treated with amikacin for seven days. Of the remainder, two patients had significant microscopic pyuria, while four did not. Urine cultures in the two patients with pyuria showed *Klebsiella spp* in one patient (patient 2; treated with nitrofurantoin on ambulatory basis). The third patient’s (patient 3) urine culture was contaminated, and a repeat culture was sterile. Since his LUTS improved significantly with increased fluid intake, he was not treated with antibiotics.

The same set of 50 patients attended the three months follow-up. Of note, the three patients (patient1, 2 and 3) who had LUTS and microscopic pyuria during first follow-up visit continued to be symptomatic at this visit too, although none was febrile. They continued to have significant pyuria at this visit. While the repeat urine culture of patient 1 was sterile, patients 2 and 3 had significant bacteriuria, the organisms were different from previous cultures ( *Enterobacter spp and Enterococcus spp*, respectively). No antibiotic therapy was prescribed in these three patients at this juncture. They were advised to maintain good hydration. All three patients had significant resolution of symptoms subsequently. Details of these patients are presented in Table 2.

**Table 2.** Details of three patients who were symptomatic on follow-up.

Characteristic	Patient 1	Patient 2	Patient 3
Diabetes	Yes	No	Yes
Past history of UTI	No	No	No
Urinary retention at presentation	Yes	Yes	Yes
Hypotension at presentation	Yes	No	No
Transabdominal ultrasound	Normal	Cystitis	Cystitis
Digital rectal examination	Enlarged and tender prostate	Tender prostate	Tender prostate
Duration of antimicrobial therapy	28 days	7 days	7 days
TRUS findings during index hospitalization	Prostatic abscess	Normal	Normal
Serum PSA at admission, one month and three months follow- up, ng/mL	5.7, 0.55, 0.55	1.9, 1.1, 0.95	2.55, 1.0,1.0
Serum hs-CRP at admission and at three months follow-up, mg/dL	2.2,0.49	1.9, 0.1	3.2,0.13

UTI, urinary tract infection; TRUS, transrectal ultrasound; PSA, prostate-specific antigen; hs-CRP, high sensitivity C-reactive protein.

### Temporal trends of serum PSA and hs-CRP

**Figure 2.**
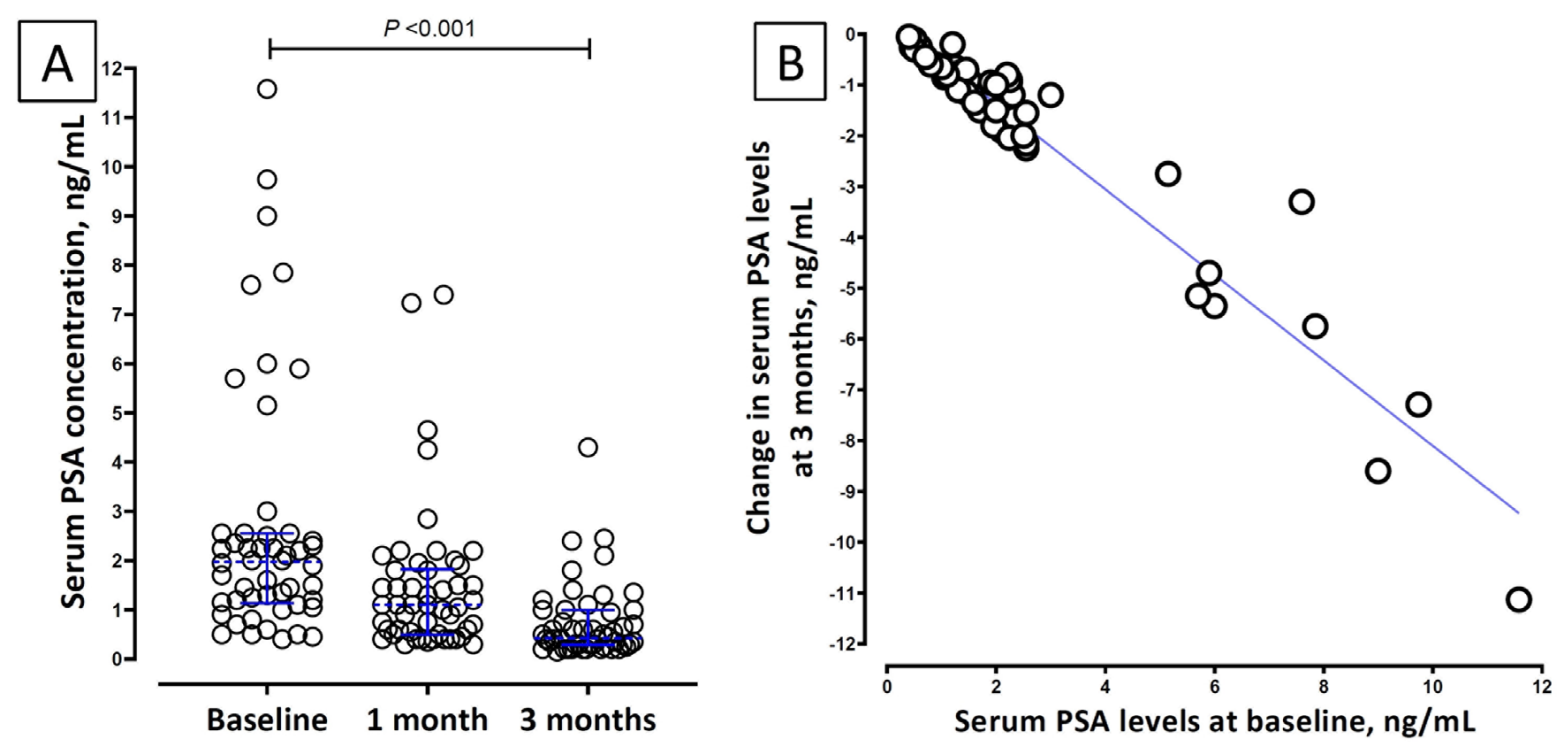
Panel **A**: Dotplot of serum prostate-specific antigen (PSA) levels at baseline, one month and three months of follow-up. Dotted blue lines across the data points depict the median and the error bars depict interquartile range. Panel **B**: Correlation between baseline serum PSA level and the change in PSA levels at three months.

At admission, 24 (38%) of 64 patients had elevated serum PSA values. Among the 50 patients who followed up, a significant decrease in serum PSA levels compared to the baseline was noticed at 3 months ( Table 3). The fall in serum PSA levels at 3 months was strongly correlated to the baseline serum PSA value (Spearman’s rho 0.93; P <0.001; Figure 2, panel B.) Of the 50 patients, 47 (94%) had prostatic involvement as per the pre-defined criteria based on significant change in serum PSA levels. This also included 29 patients whose baseline serum PSA was not elevated.

**Table 3.** Serum PSA and hs-CRP levels and TRUS findings at admission and follow-up.

Variable	All recruited patients at admission, N=64	Patients with follow-up completed, N=50			
		Admission	1 month	3 month	*P* value
Serum PSA, ng/mL, median (IQR)	2.15(1.18-3.02)	1.95(1.15-2.55)	1.1(0.5-1.8)	0.43(0.3-1)	<0.001
Serum hs-CRP, mg/dL, median(IQR)	2.23 (1.85-2.75)	2.26( 1.81-2.75)	--	0.41(0.16- 1.52)	<0.001
TRUS findings					
Prostate volume in mL, median (IQR)	25.4(18.9-34)	24.1(18.72-34.39)	--	21.6(17.7-29.3)	<0.001
Normal, n(%)	38 (59)	26(52)	--	37(74)	NA
Benign prostatic enlargement, n(%)	5(8)	3(6)		3(6)	
Focal hypoechogenicity, n(%)	6(9)	6(12)	--	5(1)	NA
Nodules, n(%)	2(3)	1(2)	--	--	NA
Abscess, n(%)	1(2)	1(2)	--	--	NA
Calcifications, n(%)	13(20)	13(26)	--	13(26)	NA
Seminal vesicle involvement, n(%)	1(2)	1(2)	--	--	NA

PSA, prostate-specific antigen; hs-CRP, high sensitivity C-reactive protein; TRUS, transrectal ultrasound; IQR, interquartile range.

**Figure 3.**
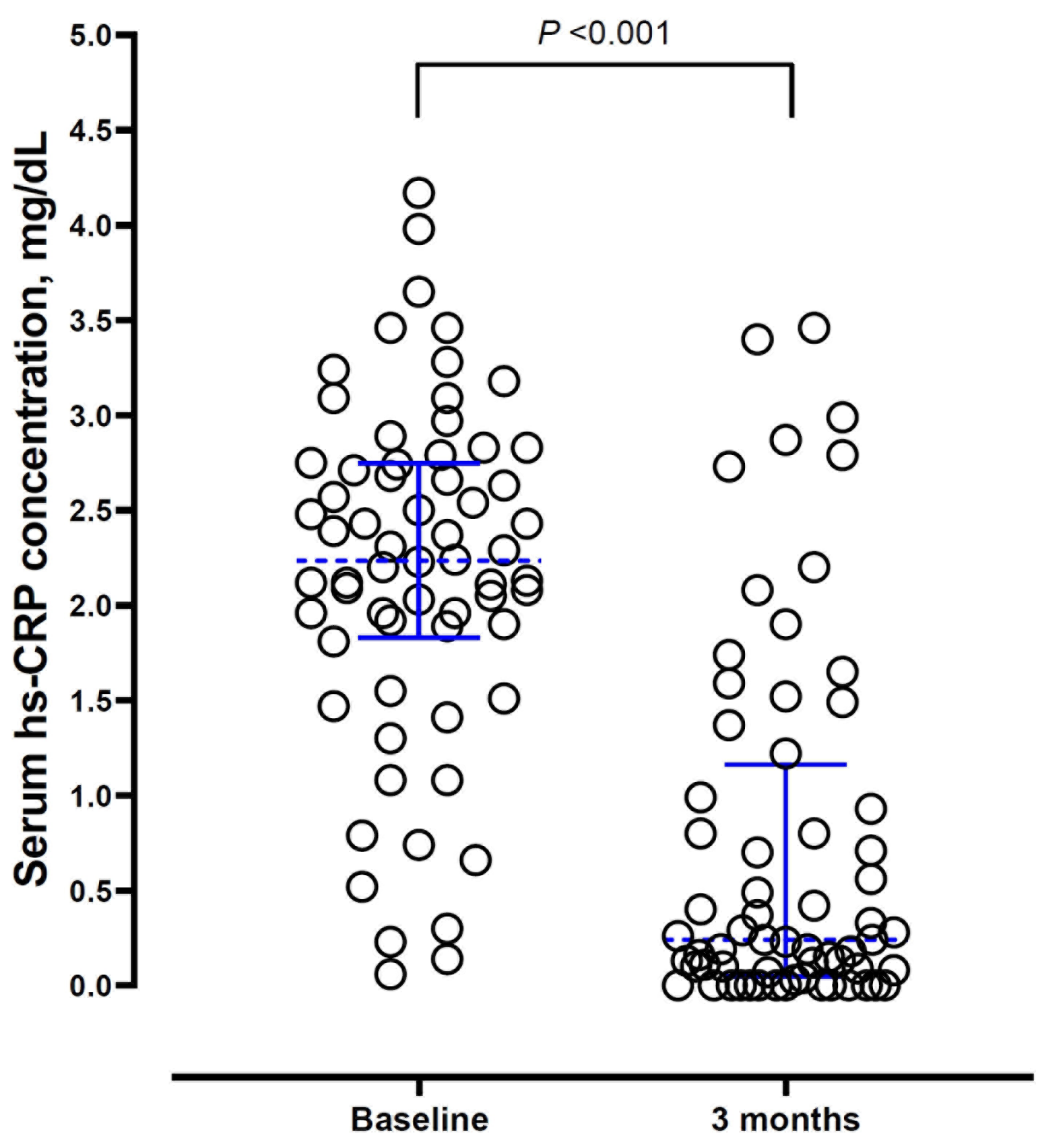
Dotplot of serum high sensitivity C-reactive protein (hs-CRP) levels at baseline and at three months. Dotted blue lines across the data points depict the median and the error bars depict interquartile range.

Serum hs-CRP levels also decreased significantly at three months compared to baseline ( Table 3, Figure 3). There was no correlation between serum PSA levels and hs-CRP levels either at baseline or at three months. The change in serum PSA levels over 3 months had no correlation with the change in serum hs-CRP levels (Spearman’s rho 0.19; *P*=0.188).

### TRUS findings during hospitalization and at three months follow-up

TRUS was done in 64 patients at baseline, within two (2–4) days of hospitalization. There were no procedure-related complications. The most significant finding was the presence of prostatic abscess in one patient. Other findings on TRUS are presented in Table 3. Follow-up TRUS examination was done in 50 patients, 95±7 days after hospital discharge. Of the six patients who had focal hypoechogenicity at admission, three continued to have the finding at three months, while new hypoechogenic lesions were noticed in two other patients. However, none of the patients who had these lesions were symptomatic at three months.

A decrease of in prostatic volume ≥10% was observed in 24 (48%) patients. The change in serum PSA levels at three months did not correlate with change in prostatic volume.

### Association of baseline and change in serum PSA levels by three months with clinical features

Serum PSA levels at baseline did not differ significantly between patients with/without clinical features suggestive of prostatic involvement such as urinary retention, lower abdominal pain, prostatic tenderness on DRE or a possible diagnosis of ABP. Similarly, none of these clinical features were associated with the change in serum PSA levels compared to the baseline ( Table 4).

**Table 4.** Comparison of baseline and change in PSA levels at three months in patients with and without clinical features of prostatic involvement.

Clinical finding	Baseline serum PSA level, ng/mL		*P* value	Change in serum PSA level at three months, ng/mL		*P* value
	Present	Absent		Present	Absent	
Urinary retention	2.07 (1.45 to 4.75)	2.15 (1.03 to 2.9)	0.40	-1.35 (-2.04 to -0.9)	-1 (-0.2 to -0.65)	0.51
Lower abdominal pain	2.0 (1.05 to 3.05)	2.4 (1.45 to 3)	0.29	-0.95 (-2.0 to -0.65)	-1.2 (-2.25 to -1.1)	0.91
Prostatic tenderness	2.1 (1.05 to2.7)	2.2 (1.2 to 4.9)	0.63	-1.35 (-2.07 to -0.6)	-1.05 (-2 to -0.7)	0.96
Possible ABP ^a^	2.2 (1.57 to 4.2)	2.15 (1.13 to 3.02)	0.61	-1.5 (-4.7 to -0.95)	-1.05 (-0.2 to - 0.67)	0.39

All data presented as median (IQR), ^a^Defined as presence of fever, dysuria, urinary retention and prostatic tenderness on DRE PSA, prostate-specific antigen; ABP, acute bacterial prostatitis; IQR, interquartile range; DRE, digital rectal examination

## Discussion

We found that about a third of men with fUTI requiring hospitalization had elevated serum PSA levels at presentation, and most men with fUTI requiring hospitalization had at least 25% decrease in serum PSA level at 3 months. Further, nearly half of them had a decrease in prostatic volume on follow up. However, only a handful of them had clinical findings suggestive of prostatic involvement, and recurrence following treatment was uncommon.

Our findings are in agreement with the seminal study by Ulleryd *et al.* ^3^. Although there are a few more studies on PSA levels in men with fUTI, these studies had enrolled patients with ABP ^11, 12^. The median (IQR) serum PSA levels at admission in our patients was 2.15 (1.18–3.02) ng/mL, which is lower compared to the study by Ulleryd *et al.*, which was 14 (range 0.54–140) ng/mL. We used a chemiluminescent immunoassay method, while Ulleryd *et al.* used a monoclonal fluoroimmunoassay. While mild assay-related variations are possible, the main reason for lower PSA levels in our study could be because of ethnic variations in PSA levels. Studies from India show that the mean serum PSA values in Indian men are lower compared to the Western population ^13– 15^.

Conventionally, elevated PSA levels and a change in prostatic volume have been proposed as definitive evidence of prostatic involvement in men with fUTI ^3, 6^. Based on this premise, often it is contended that fUTI in men should be treated with antibiotics for a duration of at least two weeks. However, interpreting changes in PSA levels and prostatic volume as reliable evidence of ‘prostatitis’ is questionable. ABP is a clinically defined entity classically characterized by fever, systemic symptoms, pelvic pain and urinary tract symptoms such as dysuria, urinary frequency, and urinary retention ^16^. Although it is known that PSA levels become elevated in men with ABP, the converse may not be true. Our findings indicate that it might be fallacious to equate pauci-symptomatic elevation of serum PSA levels as definitive evidence of prostatitis due to following reasons.

First, only a minority of patients with such changes actually had clinical findings to suggest prostatic involvement, and the PSA levels and prostatic volume changes were not related to prostatic symptoms. Second, we did not find a correlation between hs-CRP levels and the elevation in PSA levels and the change in prostatic volume. Notably, Ulleryd *et al.* also did not find a correlation between elevated serum PSA and markers of systemic inflammation. Third, despite the fact that 80% of patients were treated with antibiotics for seven days duration, recurrence of UTI was uncommon. Thus, while we confirm the findings that transient elevations in PSA levels and prostatic volume are very common in men with fUTI, we disagree with the interpretation of the clinical significance of these subclinical changes. It is quite possible that the elevated PSA levels indicate a physiological response to bacterial infection rather than indicating a pathological disease process. Townes *et al.* found that epithelial expression and release of PSA was increased by *E. coli* challenge ^17^. A few other studies also show that elevated PSA levels represent an enhanced prostate innate host defence ^18, 19^. Serum PSA levels also rise during episodes of sexually transmitted infections and may remain elevated for several months after effective antibiotic therapy ^20^. Other non-genitourinary infections like infectious mononucleosis and chikungunya also cause elevated serum PSA levels ^21, 22^. It is possible that elevated PSA level is a non-specific response to systemic inflammation caused by prostate cell damage and increased vascular permeability.

We found the presence of focal hypoechoic areas in prostate in a small proportion of patients, the significance of which is unclear — these patients also had good response to treatment and none had LUTS on follow up. Horcadaja *et al.* observed hypoechoic lesions in about 25% of patients with ABP ^23^; these lesions persisted in about one-third of patients after antibiotic therapy for one month.

The practical application of clinically distinguishing fUTI from ABP is mainly to decide on the choice and duration of antibiotics. It would be fallacious to justify the need for prolonging the antibiotic treatment based solely on biochemical and TRUS changes which might possibly suggest prostatic involvement. While clinicians generally agree on the need to treat patients with ABP for at least two weeks, it needs to be pointed out that this duration is not based on good quality evidence ^24^. In addition, considerable heterogeneity exists in the diagnosis and management of ABP among various clinical departments ^25^. Even though a shorter duration of antibiotics was associated with an increased risk of recurrent prostatitis in observational studies, it could be because those patients had clinically manifest ABP and not just biochemical and/or ultrasonographic changes ^26^. Indeed, even in ABP, some experts believe that the role of shorter treatment duration needs to be explored ^27^. This is a very important aspect since longer treatment durations have been paradoxically associated with increased late recurrences of UTI in the outpatient setting ^28^. In addition, longer antibiotic treatment durations do not augur well with the principles of antibiotic stewardship ^29^.

Very few clinical trials have addressed the issue of optimal treatment duration for fUTI in men without features of ABP. A recent trial from the Netherlands found that shorter duration resulted in lower clinical cure rates at short term in men ^30^. Prostatic involvement was attributed as a possible reason for this. However, clinical cure at 70–84 days did not differ between genders, and shorter treatment did not result in more recurrence in men on long term. Another smaller trial from India comparing non-fluoroquinolone antibiotic therapy for seven or 14 days found no difference in re-treatment rates between males and females ^31^. Average antibiotic treatment duration in the present study was less than 10 days. Yet, we did not find significant short-term recurrence of fUTI in them.

Possible limitations of our study are: i) it would have been more informative if we had longer follow-up and assessment for chronic prostatitis in the study population; ii) measurement of prostatic volumes potentially could have been affected by inter-observer variability; and iii) we did not collect data on glandular vascularity, which could indicate the presence of inflammation ^32^.

## Conclusions

In conclusion, increase in serum PSA levels and certain ultrasonographic findings, which might possibly indicate subclinical prostatic involvement, were common among men with fUTI. However, the clinical significance of these changes is uncertain.

## Data availability

### Underlying data

Figshare: Prostatic involvement in male UTI. https://doi.org/10.6084/m9.figshare.12286865.v2 ^33^

Data are available under the terms of the Creative Commons Zero "No rights reserved" data waiver (CC0 1.0 Public domain dedication).
