# Supplementary material for: Frequency and clinical significance of prostatic involvement in men with febrile urinary tract infection: a prospective observational study
Source: F1000Res. 2020 Nov 10;9:617. Originally published 2020 Jun 16. [Version 3] doi: 10.12688/f1000research.24094.3 (PMC7684675; doi:10.12688/f1000research.24094.3)
Supplement: Peer review history

# Peer review: Reviewer response for version 3

**DOI:** 10.5256/f1000research.30628.r74492

**Published:** 2020-11-23

**Authors:** Aneesh Basheer

**Affiliations:** 1 Departments of General Medicine and Medical Education, Pondicherry Institute of Medical Sciences, Pondicherry, Pondicherry, India; 2 Cochrane Neuromuscular Diseases group, London, UK

The authors have done extremely well to modify the article appropriately to address the concerns raised in previous review. 

 Congratulations on a very useful paper.

Is the work clearly and accurately presented and does it cite the current literature?

Yes

If applicable, is the statistical analysis and its interpretation appropriate?

Partly

Are all the source data underlying the results available to ensure full reproducibility?

Yes

Is the study design appropriate and is the work technically sound?

Partly

Are the conclusions drawn adequately supported by the results?

Yes

Are sufficient details of methods and analysis provided to allow replication by others?

Yes

Reviewer Expertise:

Infectious diseases, tropical medicine, hematology, evidence based medicine, medical education

I confirm that I have read this submission and believe that I have an appropriate level of expertise to confirm that it is of an acceptable scientific standard.

# Peer review: Reviewer response for version 2

**DOI:** 10.5256/f1000research.30053.r73747

**Published:** 2020-11-03

**Authors:** Aneesh Basheer

**Affiliations:** 1 Departments of General Medicine and Medical Education, Pondicherry Institute of Medical Sciences, Pondicherry, Pondicherry, India; 2 Cochrane Neuromuscular Diseases group, London, UK

Many thanks for responding patiently to my comments. 

 The revised version has more clarity of methods and the primary research question. 

 The issue of age related variability of PSA levels has also been addressed satisfactorily. 

 However, the discussion still begins with a statement that it is difficult to accept based on data presented. It states that "We found that most men with fUTI requiring hospitalization had elevated serum PSA levels and nearly half of them had a decrease in prostatic volume on follow up", although in the response to review comments the authors mention that some modification has been made. 

 Even if the said modification is considered, it would be difficult to accept the explanation of high PSA levels based on a significant fall in levels at 3 months. I would strongly urge authors to interpret the baseline PSA as it is, while mentioning that there was a significant drop at 3 months. I am disinclined to believe that such drop is indirect evidence of a high baseline PSA when actually the data pretty obviously shows PSA levels well within age specific cut offs with some outliers. 

 Apart from this I find the revised paper high in validity and methodological soundness.

Is the work clearly and accurately presented and does it cite the current literature?

Yes

If applicable, is the statistical analysis and its interpretation appropriate?

Partly

Are all the source data underlying the results available to ensure full reproducibility?

Yes

Is the study design appropriate and is the work technically sound?

Partly

Are the conclusions drawn adequately supported by the results?

Yes

Are sufficient details of methods and analysis provided to allow replication by others?

Yes

Reviewer Expertise:

Infectious diseases, tropical medicine, hematology, evidence based medicine, medical education

I confirm that I have read this submission and believe that I have an appropriate level of expertise to confirm that it is of an acceptable scientific standard, however I have significant reservations, as outlined above.

# Response

**Published:** 2020-11-04

**Authors:** Surendran Deepanjali

**Affiliations:** Jawaharlal Institute of Postgraduate Medical Education and Research (JIPMER), India

**Version 2: Response to Dr Aneesh Basheer**

 We thank Dr. Basheer for finding the revisions satisfactory, except for the beginning statement in the Discussion section. We agree with Dr Basheer that the statement that “most men with fUTI requiring hospitalization had elevated serum PSA levels” might not be factually accurate. We are happy to modify the first sentence in Discussion section as “We found that about a third of men with fUTI requiring hospitalization had elevated serum PSA levels at presentation, and most men with fUTI requiring hospitalization had at least 25% decrease in serum PSA level at 3 months.” We hope that Dr. Basheer finds this modification satisfactory.

 Thank you.

# Peer review: Reviewer response for version 2

**DOI:** 10.5256/f1000research.30053.r73746

**Published:** 2020-10-28

**Authors:** Veeravan Lekskulchai

**Affiliations:** 1 Department of Pathology, Faculty of Medicine, Srinakharinwirot University, Bangkok, Thailand

The authors have corrected their article and answered my question clearly. I have no further comments to make.

Is the work clearly and accurately presented and does it cite the current literature?

Partly

If applicable, is the statistical analysis and its interpretation appropriate?

Partly

Are all the source data underlying the results available to ensure full reproducibility?

Yes

Is the study design appropriate and is the work technically sound?

Yes

Are the conclusions drawn adequately supported by the results?

Yes

Are sufficient details of methods and analysis provided to allow replication by others?

Yes

Reviewer Expertise:

Clinical Pathology, Clinical Chemistry, Clinical Toxicology

I confirm that I have read this submission and believe that I have an appropriate level of expertise to confirm that it is of an acceptable scientific standard.

# Peer review: Reviewer response for version 1

**DOI:** 10.5256/f1000research.26580.r68790

**Published:** 2020-08-17

**Authors:** Aneesh Basheer

**Affiliations:** 1 Departments of General Medicine and Medical Education, Pondicherry Institute of Medical Sciences, Pondicherry, Pondicherry, India; 2 Cochrane Neuromuscular Diseases group, London, UK

**Rationale and research question**

 The authors submit evidence from literature that supports involvement of the prostate in a high proportion of men with febrile UTI. These studies primarily used surrogate markers to identify prostate involvement such as elevated prostate specific antigen (PSA) or increased prostate volume by ultrasonography. When acute bacterial prostatitis (ABP) is diagnosed, treatment with appropriate antibiotics for 2 to 4 weeks would be needed. ABP typically presents with pelvic pain and voiding symptoms. If the prostatic involvement in febrile UTI is a subclinical ABP, this would need similar treatment. 

 The authors tried to determine whether this subclinical involvement (evidenced by elevated PSA and prostate volume) had any association with short term UTI recurrence and clinical findings. 

 The research question is not specified anywhere in the paper. It is suggested that a clear research question be added at the end of the introduction. 

**Methods:**

 The authors have used a prospective cohort design to address the issue. This is generally the ideal method to study and correlate variables longitudinally. The methods section is well written and describes the recruitment and follow up in great detail. 

 The absence of an a-priori comparison group is a weakness, albeit minor. Instead the authors chose to analyze sub-groups from the single cohort of men with febrile UTI – those with features suggestive of clinical prostatitis and those without. A much better and internally valid design would have been the recruitment and follow up of 2 different groups in a similar manner. 

**Results:**

 The introductory paragraph on results has data pertaining to calculations based on 64 original participants, 50 finally available cases and a subgroup of cases where cultures were taken prior to antibiotics. The percentages provided therefore could confuse readers and hence it is suggested that authors clearly reframe the sentences to indicate the population from which these numbers and percentages were obtained. 

 There were 25 patients with prostate tenderness; however, authors mention that only 8 could be classified as Acute bacterial prostatitis. It is not clear then under what banner, the remaining 17 patients fall? 

**Discussion:**

 The authors state that most men with febrile UTI had elevated PSA. From the results it is not clear what cut-off was used to determine elevated PSA. Since PSA cut offs are generally age based, it would also be interesting to note the effect of age of the cases on the PSA levels. A regression analysis could probably provide useful information in this regard. Moreover, the results section states that only 14 (22%) of the participants had PSA levels more than 4 ng/mL. The median PSA of the patients was 2.15 which is also well within normal ranges for the lowest age groups used for PSA cut offs. This disparity between results and the discussion section needs to be clarified. 

 Authors noted a statistically significant drop in the PSA levels at baseline and at 3 months. Further, 94% of patients had more than 25% drop which was the predefined significant change.

 The predefined significant change occurred. However, the baseline PSA itself was not high. The authors need to discuss this in their limitations. It is possible that the 25% reduction considered by authors may not be the “minimal clinically important difference”. The other possibility is that these effects have been affected by the low baseline prostate involvement that was presupposed as 80% for sample size calculation. 

**Overall comments**: Well written except for minor modifications suggested.

Is the work clearly and accurately presented and does it cite the current literature?

Yes

If applicable, is the statistical analysis and its interpretation appropriate?

Partly

Are all the source data underlying the results available to ensure full reproducibility?

Yes

Is the study design appropriate and is the work technically sound?

Partly

Are the conclusions drawn adequately supported by the results?

Yes

Are sufficient details of methods and analysis provided to allow replication by others?

Yes

Reviewer Expertise:

Infectious diseases, tropical medicine, hematology, evidence based medicine, medical education

I confirm that I have read this submission and believe that I have an appropriate level of expertise to confirm that it is of an acceptable scientific standard, however I have significant reservations, as outlined above.

# Response

**Published:** 2020-10-03

**Authors:** Surendran Deepanjali

**Affiliations:** Jawaharlal Institute of Postgraduate Medical Education and Research (JIPMER), India

**Response to Dr Aneesh Basheer**

We thank Dr Basheer for the very constructive comments, and we have modified the manuscript incorporating his suggestions. Please find below a point-by-point response to Dr Basheer’s comments and queries.

**Comment:** *Rationale and research question. The authors submit evidence from literature that supports involvement of the prostate in a high proportion of men with febrile UTI. These studies primarily used surrogate markers to identify prostate involvement such as elevated prostate specific antigen (PSA) or increased prostate volume by ultrasonography. When acute bacterial prostatitis (ABP) is diagnosed, treatment with appropriate antibiotics for 2 to 4 weeks would be needed. ABP typically presents with pelvic pain and voiding symptoms. If the prostatic involvement in febrile UTI is a subclinical ABP, this would need similar treatment. The authors tried to determine whether this subclinical involvement (evidenced by elevated PSA and prostate volume) had any association with short term UTI recurrence and clinical findings. The research question is not specified anywhere in the paper. It is suggested that a clear research question be added at the end of the introduction. The research question is not specified anywhere in the paper. It is suggested that a clear research question be added at the end of the introduction.*

**Response:** We thank Dr Basheer for summing up our study’s rationale very thoroughly. As per your suggestion, we have now explicitly spelt out the aim of our study at the end of Introduction. We write  “ We, therefore, conducted the present study to answer the following research questions — i) What is the frequency of prostatic involvement in men with fUTI, and ii) Is prostatic involvement associated with recurrence of UTI?”

**Comment: **Methods **:** *The authors have used a prospective cohort design to address the issue. This is generally the ideal method to study and correlate variables longitudinally. The methods section is well written and describes the recruitment and follow up in great detail.*

**Response:** We thank Dr Basheer for the encouraging comments.

**Comment:** *The absence of an a-priori comparison group is a weakness, albeit minor. Instead the authors chose to analyze sub-groups from the single cohort of men with febrile UTI – those with features suggestive of clinical prostatitis and those without. A much better and internally valid design would have been the recruitment and follow up of 2 different groups in a similar manner.*

**Response: **We would like to clarify that the definition of prostatic involvement was specified *a priori* in our study protocol. We had given in the Methods section of version 1 that “As defined by Ulleryd et al [3], a fall of ≥25% in serum PSA levels at 3 months, and a decrease in prostatic volume of ≥10% at 3 months were considered significant.”  However, for the sake of better understating, we have re-worded this as “We defined prostatic involvement as per the criteria suggested by Ulleryd et al [3]. A reduction of serum PSA by >25% irrespective of the initial PSA level, and/or a decrease in prostatic volume by >10% after 3 months was taken as evidence of prostatic involvement.” By nature of this definition, it is not possible to classify patients upfront at inception into those with and without prostatic involvement, as suggested by Dr Basheer. However, we agree with Dr Basheer that these patients could be followed up beyond 3 months to ascertain long term outcomes, which was not done in our study.

**Comment: **Results: *The introductory paragraph on results has data pertaining to calculations based on 64 original participants, 50 finally available cases and a subgroup of cases where cultures were taken prior to antibiotics. The percentages provided therefore could confuse readers and hence it is suggested that authors clearly reframe the sentences to indicate the population from which these numbers and percentages were obtained*.

**Response: **We thank Dr. Basheer for pointing this out. We have now modified the introductory paragraph taking care that the reference population for the percentages is clearly specified. Also, data presented in Table 3 now clearly demarcate information from all 64 recruited patients at baseline from those 50 in whom complete follow-up is available. Similarly Figures 2 & 3 have been modified to represent data from 50 patients only.

**Comment:** *There were 25 patients with prostate tenderness; however, authors mention that only 8 could be classified as Acute bacterial prostatitis. It is not clear then under what banner, the remaining 17 patients fall?*

**Response: A**s we had pointed out in the Discussion section, it is known that there is much heterogeneity among clinicians from different disciplines in arriving at a diagnosis of ABP (Reference 24, version 1). While some clinicians might feel that prostatic tenderness alone is sufficient to diagnose ABP in men with fUTI, many physicians might not agree with this. Therefore, to ensure reproducibility we have adopted a classical syndromic definition for ABP in which only those patients with tender prostate as well as urinary retention (which is a voiding symptom) were classified as ABP. We chose this stricter definition to make the clinical diagnosis unambiguous, since we also intended to evaluate whether the diagnosis had any bearing on baseline serum PSA levels and its longitudinal changes (Table 4, version1). While it is quite possible that the remaining 17 patients had a *forme fruste* of ABP, the present study was not designed to answer this question. Moreover, even when the analysis was based on individual symptoms, we did not find any association with serum PSA levels (Table 4).

**Comment: **Discussion: *The authors state that most men with febrile UTI had elevated PSA. From the results it is not clear what cut-off was used to determine elevated PSA. Since PSA cut offs are generally age based, it*

*would also be interesting to note the effect of age of the cases on the PSA levels. A regression*

*analysis could probably provide useful information in this regard. Moreover, the results section*

*states that only 14 (22%) of the participants had PSA levels more than 4 ng/mL. The median PSA of*

*the patients was 2.15 which is also well within normal ranges for the lowest age groups used for*

*PSA cut offs. This disparity between results and the discussion section needs to be clarified.*

*Authors noted a statistically significant drop in the PSA levels at baseline and at 3 months. Further,*

*94% of patients had more than 25% drop which was the predefined significant change.*

*The predefined significant change occurred. However, the baseline PSA itself was not high. The*

*authors need to discuss this in their limitations. It is possible that the 25% reduction considered by*

*authors may not be the “minimal clinically important difference”. The other possibility is that these*

*effects have been affected by the low baseline prostate involvement that was presupposed as 80%*

*for sample size calculation.*

**Response: **We thank Dr Basheer for drawing our attention to this important aspect of interpretation of serum PSA levels in our study population.  We would like to clarify that we stated that serum PSA levels were elevated in most men with febrile UTI, based on the observation that almost 94% of men had a significant fall in serum PSA values at 3 months compared to their baseline value. Following Dr Basheer’s suggestion, we have incorporated age-specific cut offs in the revised manuscript to interpret the PSA values at baseline (Please see last paragraph under ‘Study procedure’ in Version2). It is true that only 38% of patients had a baseline PSA value above the upper limit of their age-specific reference age. We use the 97.5 ^th^ percentile value as the upper limit of reference range. However, serum PSA values in apparently normal healthy men in a specific age-group could show considerable variability within the reference range. For example, in the age-group 50-59 years the minimum value was 0.06 ng/mL, while the maximum was 5.9 ng/mL. Thus, even if the baseline value was within the reference range, a dynamic fall on follow-up would suggest that the baseline was elevated for a given patient.

Nevertheless, we agree that the statement “We found that most men with fUTI requiring hospitalization had elevated serum PSA levels” could be confusing, and hence we have modified this in Version 2. We have now modified it as “We found that most men with fUTI requiring hospitalization had significant decrease in serum PSA levels at 3 months follow-up indicating an elevated baseline value, and nearly a half of them had a decrease in prostatic volume on follow-up.”

As suggested by Dr Basheer, we checked for a linear relationship between age and baseline PSA levels by doing a simple linear regression. However, we did not find a relationship between age and PSA levels (coefficient = 0.017; P = 0.486). Although it is well known that PSA levels increase with age, we did not find such a relationship. Most probably, infection-induced changes in PSA distort and overshadow any such underlying relationship in our dataset. Similar to our findings, Ulleryd et al (reference 3) also did not find a correlation between age and serum PSA levels during episode of fUTI in men.

We hope Dr Basheer finds the modifications satisfactory, and we once again express our gratitude for your suggestions. We would definitely address any other ensuing concerns regarding our manuscript.

Thank you.

# Peer review: Reviewer response for version 1

**DOI:** 10.5256/f1000research.26580.r68741

**Published:** 2020-08-10

**Authors:** Veeravan Lekskulchai

**Affiliations:** 1 Department of Pathology, Faculty of Medicine, Srinakharinwirot University, Bangkok, Thailand

This work is entitled "Frequency and clinical significance of prostatic involvement in men with febrile urinary tract infection: a prospective observational study" and was written by Arjunlal *et al. *The authors studied chances of having acute bacterial prostatitis in male admitted with urinary tract infection. The manuscript was written appropriately and relevantly. However, I have a few major concerns for this work. First in the Results, though, 64 patients were enrolled and their baseline results were available, 14 of them were not followed up and were excluded after that. Consequently, the baseline results of these 14 patients should be excluded from this work (Table 3, Figure 3). The baseline results should come from 50 patients similar to the results in the next one and 3 months. Another concern is why they used hs-CRP in cases whose inflammation were obviously indicated. They should use CRP level. This point needs an explanation.My minor concern is the use of old references. If possible, they should be replaced by newer ones.

Is the work clearly and accurately presented and does it cite the current literature?

Partly

If applicable, is the statistical analysis and its interpretation appropriate?

Partly

Are all the source data underlying the results available to ensure full reproducibility?

Yes

Is the study design appropriate and is the work technically sound?

Yes

Are the conclusions drawn adequately supported by the results?

Yes

Are sufficient details of methods and analysis provided to allow replication by others?

Yes

Reviewer Expertise:

Clinical Pathology, Clinical Chemistry, Clinical Toxicology

I confirm that I have read this submission and believe that I have an appropriate level of expertise to confirm that it is of an acceptable scientific standard, however I have significant reservations, as outlined above.

# Response

**Published:** 2020-10-03

**Authors:** Surendran Deepanjali

**Affiliations:** Jawaharlal Institute of Postgraduate Medical Education and Research (JIPMER), India

**Response to Dr. Veeravan Lekskulchai**

 We thank Dr Lekskulchai for the very helpful and constructive comments. We have tried to address the concerns raised by Dr Lekskulchai and have made modifications in our manuscript accordingly. Please find below a point-by-point response to the comments.

**Comment:** *This work is entitled "Frequency and clinical significance of prostatic involvement in men with*

*febrile urinary tract infection: a prospective observational study" and was written by Arjunlal* *et al.*

*The authors studied chances of having acute bacterial prostatitis in male admitted with urinary*

*tract infection. The manuscript was written appropriately and relevantly. However, I have a few*

*major concerns for this work.*

**Response: **We are grateful for the kind compliments. We hope to address the concerns raised by Dr Lekskulchai.

**Comment:** *First in the Results, though, 64 patients were enrolled and their baseline results were available, 14 of them were not followed up and were excluded after that. Consequently, the baseline results of these 14 patients should be excluded from this work (Table 3, Figure 3). The baseline results should come from 50 patients similar to the results in the next one and 3 months.*

**Response: **We than Dr Lekskulchai for pointing this out. We have now presented the important baseline variables pertaining to the 50 patients separate from the total 64 recruited patients in Table 3. Also, we have modified Figure 3 accordingly to include data pertaining to these 50 patients only.

**Comment** *:  Another concern is why they used hs-CRP in cases whose inflammation were obviously indicated. They should use CRP level. This point needs an explanation.*

**Response: **Dr Lekskulchai is correct in pointing out that serum CRP estimation would be better than hs-CRP in patients with obvious inflammation such as febrile UTI. However, a pre-specified follow-up at 3 months was planned in our study, and it was expected that UTI-associated inflammation would have substantially decreased in many patients on follow-up. Since a more sensitive assay would be required to demonstrate this low-grade inflammation with CRP concentrations often below 1.0 mg/dL, we used an hs-CRP assay rather than a CRP assay. Previous studies on subclinical prostatic inflammation have used hsCRP as a biomarker ( *Milbrandt M, Winter AC, Nevin RL, et al. Insight into infection-mediated prostate damage: Contrasting patterns of C-reactive protein and prostate-specific antigen levels during infection. Prostate. 2017;77:1325-1334.*).

 We are also duty-bound to inform Dr Lekskulchai that the query on the utility of hs-CRP made us look closely at the hs-CRP primary data.  We then identified an inadvertent data entry error. We realized that the optical density values of the ELISA read-outs were mistakenly entered as the actual hs-CRP values. We sincerely regret this inadvertent error. We have now uploaded a corrected version of the underlying data set containing the corrected hs-CRP values which can be found at *Deepanjali, Surendran; Thayyil, Arjunlal; Rajappa, Medha; Ramanitharan, Manikandan (2020): Prostatic involvement in male UTI. figshare. Dataset.* *https://doi.org/10.6084/m9.figshare.12286865.v3* *. * Accordingly, we also have now made necessary changes in descriptive data on serum hs-CRP as well as Figure 4.  While this error does not change our findings and conclusions, we found that the weak correlation between change in serum PSA levels with change in serum hs-CRP levels was no longer significant when the analysis was repeated using corrected hs-CRP values. We have now provided this information in Version 2.

**Comment:** *My minor concern is the use of old references. If possible, they should be replaced by newer ones.*

**Response: **We ran an updated PubMed search using terms “serum PSA AND urinary tract infections”, “serum PSA and acute bacterial prostatitis” and “hs-CRP AND urinary tract infection”. However, we could not find any newer reference related to our manuscript. If Dr Lekskulchai could kindly point out any particular reference/references which needs to be checked for new information, we would be happy to do so.

 We sincerely hope Dr Lekskulchai finds our responses satisfactory. We would certainly try to address any further concerns if present.

 Thank you.
